# Commodifying snow, taming the waters. Socio-ecological niche construction in an Alpine village

**DOI:** 10.1007/s12685-015-0123-0

**Published:** 2015-05-13

**Authors:** Robert Gross, Verena Winiwarter

**Affiliations:** Institute of Social Ecology/Center for Environmental History, Alpen-Adria-University Klagenfurt, Schottenfeldgasse 29, 1070 Vienna, Austria

**Keywords:** Human niche construction, Socio-ecological niche construction, Environmental history, Winter tourism, Artificial snow systems

## Abstract

White belts of snow clad mountains all over the world each winter. Even if there is no snow, the tourism industry is able to produce the white finery at the push of the button, thereby consuming large amounts of water. Studying Damüls, a well-known ski resort in Austria’s westernmost province Vorarlberg, we can show that the development of a service sector within agro-pastoral landscapes was connected with novel water uses and massive interventions into Alpine landscapes. Human niche construction theory offers a unique avenue for studying the development of Alpine communities, but also highlights side effects accompanying the change from agrarian to tourism livelihoods. One aim of this paper is to broaden the scope of human niche construction theory. Inceptive, counteractive and relocational niche construction activities were coupled to the differentiation of actor groups. To incorporate social dynamics, indispensable for studies in environmental history, we propose the concept of socio-ecological niche construction. The paper investigates how villagers balanced resource limitations typical for an agrarian society with the differentiation of sub-niches, mediating selective forces on the population. When the valleys were industrialized, Damüls was almost given up as a permanent settlement. Then, tourists entered the stage, by and by turning the wheel of local development into a different direction. A tourism niche based on natural snow evolved from the 1930s onwards. While the socio-ecological niches of agriculture and tourism coexisted in the interwar years, this changed when ski lifts were built, embedded into a debt-based economy that made the tourism niche vulnerable to snow availability. Snow-dependency became a powerful selective force. It was mediated by the ski lift companies through a range of niche construction activities that turned water into an important resource of snowmaking systems.

## Introduction

The study area Damüls is a small village in the westernmost part of Austria, located in the province of Vorarlberg. It was settled in the thirteenth century as a mainly pastoral hamlet; local subsistence rested on combining transport services with the use of mountain meadows through livestock. The village area is 20,9 km^2^, located on altitudes from 1,432 to 2,000 meters above sea level. Damüls receives plenty precipitation (1,400–1,500 mm/year). From mid-October to the end of April the area is covered by up to 1.5–2 m of snow. Dairy farming on meadows and pastures is reduced to a very short period of 150–180 days (Staudinger 2009). Due to these harsh surroundings no agricultural intensification was possible in Damüls. By 1924, the village was at its all-time population minimum. The rise of winter tourism paved the way (quite literally) for population increase, social change and a new way of using the local environment for ski routes and ski lifts. Latest developments include the building of storage ponds for water to be used for artificial snowmaking. Artificial snowmaking has created new types of environments in which new species, adapted to these environments, thrive. A new species of algae, *Chloromona Skiensis* is an unwanted and unplanned side-effect of human intervention into Alpine ecosystems (Hoham et al. 1993). How can this development be understood? Based on original research by the first author, we chose niche construction as a concept to narrate the environmental history of this Damüls.

Environmental historians have in the past chosen from several narratives when emplotting a particular case they study. Human niche construction offers a conceptual toolkit to integrate the perspective of biologists as well as of historians, as demanded by Edmund Russell (2011). In this paper, we shall use human niche construction as meta-narrative within which a temporally and causally coherent plot about winter tourism development in an Alpine village is constructed.

In ecology, niches were originally regarded as features of the environment and later conceptualized as extroversion of species’ requirements (Mayr 2003, S. 53) Human ecologist Donald L. Hardesty proposed to use the “Hutchinsonian concept of the ecological niche”, understanding ecological niches as a characteristic of the species, not of the environment, by defining the ecological niche “in terms of the distinctive ways of using resources for subsistence that set ‘cultural species’ apart” (Hardesty 1975). In other words, populations of humans are socially differentiated into groups of actors sharing more similar interests, perceptions and livelihoods than they share with other actor groups.

The niche construction perspective bridges the dichotomy of the original concepts by emphasizing the active role of species, which modify existing selective pressures, thereby enhancing their reproductive success (Day et al. 2003). In doing so, they change the environment in which they and others grow, develop, and learn. This frequently proceeds in ways that change the pattern of natural selection, acting back on the population as well as on other species that cohabit their niche. Among the many effects of niche construction Laland et al. have identified, one is of particular importance here. Niche construction affects carrying capacities, species’ diversity, robustness and macro-evolutionary trends. (Laland et al. 2013). In this paper, water, both liquid and frozen, is discussed as a key resource, sometimes limiting or fostering but always affecting human’s reproductive success in an Alpine village.

As environmental historians, we are interested in niche construction, because it can generate long-term effects on ecosystems. For instance, beaver dams deteriorate without beaver activity, but the deterioration leads to meadows that can persist for decades and rarely return to the original vegetation (Hastings et al. 2007). Such legacies are known as ‘ecological inheritance’, which comprises modification of biotic and abiotic factors, bequeathed by niche-constructing organisms to descendant organisms (Laland et al. 2013). Humans exhibit an extraordinary niche construction capacity that “stems from the capacity for rapid rates of change through cultural transmission”. (Laland et al. 2014, p. 436) Environmental problems such as soil degradation, water pollution, climate change and biodiversity loss can be considered as a legacy of previous alterations of selective forces, which affected the control webs that underlie ecosystems. As Laland et al. put it, causality flows through the ecosystem, from cultural to genetic and back to cultural processes, and from one species to the next, driven by iterative bouts of niche construction and selection at multiple levels (Laland et al. 2013).

Building on the insights of Laland et al., we use niche construction theory to tell an environmental history of an Alpine village shaped by the differentiation of new actor groups and the development of a novel economic way of life—the service society—within an agrarian landscape and the implication of this niche construction activity for Alpine water systems.

 In Damüls we observe differentiation between an agrarian livelihood and other forms of economy and land use, accompanied by different types of human niche construction. Inhabitants physically changed components of their environment, e.g. by building infrastructure or transforming grassland into arable land, which can be analyzed as inceptive niche construction or as counteractive niche construction. According to Jeremy Kendal et al., it is crucial if populations react to earlier niche construction activities or not. In the first case, Kendal et al. characterize these activities as counteractive niche construction. The latter would be categorized as inceptive niche construction (Kendal et al. 2011). In our study region both forms are entangled. In contrast, relocational niche construction denotes actively migrating parts of the population (O’Brien and Laland 2012). The study of the Alpine village Damüls shows that niche construction of humans is closely connected to available physical, economical as well as symbolic resources. We therefore suggest to broaden human niche construction theory by including the study of social phenomena. To embrace the complexity of “the social”, we suggest **socio-ecological niche construction** as narrative for environmental history. This narrative has two central facets: One is the structuring effect of the landscape and material artefacts on social life and, thus, on human history, and the other one is the notion that all physical interactions between people and their environment are based on opinioned perceptions. Narrated as socio-ecological niche construction, the Alpine village Damüls appears as a dynamic selective environment for the inhabitants, which is structured by the social complex as much as it structures social complexity (Laland et al. 2014). Each generation has to deal with the ecological inheritance of its ancestors. The ecological inheritance affects the population in its reproductive success but also serves as external representation of embodied knowledge, which channels behaviour and stimulates social learning processes. (Mesoudi et al. 2013, p. 204).

## How water surplus shaped an agro-pastoral socio-ecological niche over centuries

The area which was later named Damüls was given as an “Erblehen” [entail, transl. by R.G.] to a group of people called “Walser” in 1313, who permanently settled there. The name “Damüls” is derived from Latin “mulgere” which means “milking cows” and indeed, this area was used to breed and feed cattle for centuries (Kasper 2013). When the “Walser” arrived in the area, they created an ecosystem adjusted to the needs of their livelihood. They cleared the forest, and transformed the slopes into grassland for dairy farming. A census in 1,769 counted 280 inhabitants in 63 households using 64 farm houses. Furthermore, the census counted one mason, one shoemaker and two carpenters. Most inhabitants were involved in dairy farming and cattle breeding. The inhabitants held 90 cows, 16 pieces of “Galtvieh” [cattle older than 2 years], 12 calves and one sheep permanently. Livestock number increased during the summer months. Especially poorer families temporarily held horses, cattle, calves, sheep and goats from the surrounding valleys. The number of domesticated animals doubled over summer (VLA Statistisches vor 1850). Damüls was a high alpine summer pasture in the eighteenth century for productive livestock from the valleys.

The population of Damüls was confronted with natural selective forces, resulting from a combination of high altitude, steep slopes, unstable and rainy weather conditions during the summer, as well as the destructive power of avalanches in wintertime (VLA Statistisches vor 1850). Water in all aggregate states played an important role as selective force, impairing the reproductive success in the agricultural niche. Seasonal water surplus was intrinsically tied to the agricultural niche in Damüls and as such it was taken as a given by the inhabitants. No archival sources point to drainage, erosion control or avalanche protection. Inhabitants mediated selective pressures caused by water with a range of strategies, indicating high social adaption to selective forces and social learning processes. First, as described above, the “Walser” in Damüls offered their constructed ecological inheritance and work force to cattle breeders from the valleys. By doing so, the inhabitants improved reproductive success by creation of a monetary buffer. Second, they constructed their socio-ecological niche by mobility strategies as a resource to enlarge the niche’s carrying capacity. The strongest men of Damüls transported flour and grain on a six to seven hour walk on their backs from markets in the Rhine valley to Damüls (Tiefenthaler 1950). Population manipulated the carrying capacity of its agro-pastoral niche by acquiring additional resources. Third, the inhabitants of Damüls decreased population size by temporary migration. In 1837, parish priest Joseph Hummer described temporary migration: “Die minderbemittelten senden ihre noch zarten Kinder im Sommer in andere Länder, damit diese dort ihr Geld verdienen […]. Es gibt zudem halbwüchsige, die in die Schweiz ziehen, damit sie dort mit ihrer Hände Arbeit für sich und ihre Eltern sorgen […]” (Sammlung Bruno Bischof 1837). [The poorer families send their children to other countries to earn money over the summer. Furthermore, adolescents work in Switzerland to care for themselves and their parents, transl. by R.G.]. Fourth, the local population established a local job market mainly for women. Textile manufacturers in the valleys outsourced some steps of the production process mainly to women in alpine villages, because they worked quickly, reliably and for little money. In the early decades of the nineteenth century, home-work for the textile industry supplied a large part of the population with additional income (Feuerstein 1929). Money and cultural differentiation of subgroups trained in handicrafts served in Damüls as buffer by which shrinking incomes from pasture were substituted. Fifth, inhabitants constructed an agricultural sub-niche by adopting potato cultivation. Potatoes were adapted to disadvantageous soils and high alpine climates, and are resistant to hailstorms and snowfall during the summer (McC. Netting 1981). The introduction of potato cultivation reduced the dependence on imports. A land survey of 1857 documents 226 potato plots, in total 5.9 ha in Damüls (VLA Österr. Grundkataster 1857). Using Robert McC. Netting’s numbers for the Alpine village of Törbel/Switzerland, we infer that farmers could produce between 87 and 104 tons of potatoes a year.^1^ Since about 20 % of the harvest needs to be saved as seed potatoes for the following season, we conclude that each inhabitant—including the aged and children—could consume between 500 and 600 g of potatoes per day. While these figures are estimates, local sources provide evidence that farmers in Damüls produced a surplus sold to other villages as “Damülser Vieläugler” (Feuerstein 1929). By combining these strategies, the population was able to make its living without taming water flows and snow masses, which would have been cost- and labor intensive.

Each environment possesses a finite carrying capacity at any point in time. According to niche construction theory it’s a function of the niche-constructing activity of the focal population (Gurney and Lawton 1996; Odling-Smee et al. 2014). Population statistics show that niche-construction activities were effective in Damüls. From 1754 to 1810, the population increased by 38 persons from 280 to 318 inhabitants. In 1817 the population had further grown, 352 inhabitants were counted in total, rising to 415 in 1850. (Klein 2014). Within one century, the total population had grown by about 48 %. The population constructed an agro-pastoral niche that was differentiated into different sub-niches. Each sub-niche was coupled to a specific actor group, with shared cultural presumptions concerning their socio-economic and biophysical reproduction. The anthropologist Jerome H. Barkow described this process with the following words: “As cultures differ from one another, our niches, and therefore the selection pressures to which we are subject, may differ from cultural group to cultural group” (Barkow 2000). In Damüls, the diversified agro-pastoral niche was effective over centuries as a portfolio strategy. When shifts in selective forces occurred, e.g. when climate cooling impeded productivity in the grassland niche, the transport service- and stitchery niches compensated losses and assured reproductive success of the population. Later, the potato niche reduced dependency on imports and improved the food supply. A permanent balancing of counteractive, inceptive, relocational and pertubational niche construction activities within the niche network enabled to sustain a growing population in Damüls.

The inhabitants applied these strategies over more than five centuries. But when the first coal-driven steam trains reached the adjacent Rhine valley in mid of the nineteenth century, a causal chain was set in motion, which would have a sustained impact on Alpine villages. Now, cheap imports, among others of cotton, grain and potato reached Vorarlberg. Exporting manufactured textiles became easier and much cheaper. The textile manufactories in the valleys, and with it labor demand grew considerably. (Weitensfelder 2001; Bauer 1930; Feuerstein 1929). Formerly outsourced manual stitching activities was replaced by machines. The village lost income possibilities. Their numbers decreased by about 47 % from 383 in 1880 to 204 inhabitants in 1924 (Gross 2012). The drop of population remodeled the agro-pastoral niche in Damüls. Due to the lack of available workforce, potato cultivation was given up. People again had to import the basics of their daily diet. In 1924 the strategies of the remaining population to mediate selective pressures were narrowed down to dairy farming and cattle breeding.

## Skier’s as a resource for the creation of a tourism niche

The ecological inheritance generated by “Walser” settlers, seeking new ways of diversification made possible by the new means of transportation, became more and more popular in the valleys in the course of the nineteenth century as an aesthetic pleasure. The material precipitate of the agro-pastoral niche construction was not only valued highly aesthetically, but became functional in an entirely new way. The cleared, south facing slopes and the stony mule tracks provided a niche for hikers seeking pleasure. Nevertheless, Damüls did not become particularly popular as a summer resort, due to its lacking infrastructure and shortages of consumer goods. This changed when ski sports started to boom. The village became part of the mental topography of urban skiers. The first skiers came to Damüls in 1905. In 1924 the area was already connected to a trans-regional ski trail from the Rhine valley to Lech am Arlberg, the latter being a winter tourism hotspot. However, limited transport connections, small housing capacity and shortage of capital hampered the winter touristic commodification of Damüls.

Meanwhile, crowds of war veterans who had learned to ski during WWI formed in Alpine and skier-associations. The image of skiing became considerable more prestigeous. Societal attitude to snow shifted from aversion and disinterest to enthusiasm and with it, snow became an integral resource for many Alpine communities. The opening line in Luis Trenkers (1892–1990) famous book “Berge im Schnee” [mountains covered by snow, transl. by R.G.] vividly demonstrates the coupling of skiing and a positively connoted perception of snow (Trenker 1935). “Am Anfang aller Freude und jeden Wissens um den Ski war der Schnee. Möglich, daß die Wasser der Erde zuerst aus seinem Kristall sickerten, als sich aus Dämpfen und Weltraumkälte der erste sechszackige Schneestern bildete” (Trenker 1935, p. 9). [Snow was at the beginning of all joy and knowledge about skiing. Probably all the earth’s water trickled from a crystal when vapors and universe built up the first six-spiked snowflake, transl. by R.G.] Trenker was one of the avant-garde forerunners when it came to promoting an urban-centered, cliché-laden perception of snow and the wintry Alps. However, Trenker did not only pave the way for a novel perception of snow, he taught snow-knowledge to the readers of his books. Thereby, he stimulated a social learning processes that aimed to popularize the short time exposition of human bodies to selective forces in high altitudes, which were formerly considered predominantly as inhospitable.

For Trenker skiing meant the interplay of different ways of moving human bodies—namely stepping, gliding, swinging and jumping—with skis and snow cover. Snow quality remained uncontrollable in Trenker’s time, he focused on controlling the skiers’ body (Trenker 1935, p. 15). Skiers had to learn to handle different kinds of snow by controlling the skis and their legs. The most important thing was to learn to perceive the different qualities of snow. In the first chapter of “Berge im Schnee” Trenker creates a typology of snow following its life-cycle. He starts with fresh snow that turns into sticky-snow once it lies on the ground. During cold nights, sticky snow transforms to powder and over a couple of frosty nights into granulous snow and further into frozen snow called firn (Trenker 1935, pp. 14–17).

In the next chapter, Trenker increased the complexity of the described system by adding skiers. “Wir kennen keinen Erfinder des Ski. Das Gerät gehörte wie Pfeil und Bogen, wie Bumerang, Lanze, Keule oder Rad zu den Gegenständen, die kein einzelner ersinnen konnte […]. Widerstände verschiedenster Art sind es, die den Menschen zwingen, sie durch irgendeine Form von Tätigkeit oder Hilfsmittel zu überwinden” (Trenker 1935, p. 23) [We dont know who invented skies. Like bow and arrow, boomerang or lance, cudgel or the wheel, skis could not be invented by one single person. Resistances of various types had to be overcome by activities or tools, transl. by R.G.] Trenker uses an evolutionary analogy of adaptation to selective pressures as explanation for the invention of skies. According to Trenker, humans in Nordic environments increased their reproductive success by using skies when hunting or trading (Trenker 1935, p. 24). This analogy was very common in the interwar years. It depicts the progressive and modernist thinking of many Alpinists but it also shows what sport was about in those days: Modern skiing developed in the context of a growing influence of Social Darwinism. The genetic material of national populations should be improved by controlling the reproduction of subgroups perceived as inferior. Social Darwinists also aimed to improve the population by training of the “Volkskörper” [peoples’ body, transl. by R.G.] (Müller 2009).

Ironically, the culturally constructed snow-based ski system had an impact on human populations not by eugenic or genetic evolution. The evolved capacity for cultural transmission and learning, which is a crucial element of socio-ecological niche construction, paved way to transfer the snow-based ski system into various environments, which had resulted from past niche-construction activities of farmers (Boyd and Richerson 1985; Dean et al. 2012; Rendell et al. 2011). These environments were re-named “Skiparadiese” [skiing paradises, transl. by R.G.] or “Wintersportplätze” [winter sport places, transl. by R.G.] and step by step were remodeled in their materiality. In Damüls, novel guesthouses were erected. In 1928 guesthouse “Alpenblume” was built. In 1932 “Berghotel Madlener” opened its doors, followed by “Gasthaus Sonnenheim” in 1934 and “Berggasthof Walisgaden” in 1938. Between 1931 and 1933 the number of sleeping berths in hotels and guesthouses quadrupled from 50 to 200. At the same time, farmers began to offer rooms in their farmhouses and alpine cabins. Private beds doubled between 1931 and 1933 from 50 to 100 (Gross 2012). The newly built guesthouses also provided space for novel services: A hairdresser, a photographer, a sporting goods and skiing repair shop, as well as a bakery store opened businesses in the early 1930s. Ski jumps and an ice rink replenished the constructed environment of the snow-based ski system. Last but not least, a tourist association was founded and coachmen were hired to transport tourists from the valley to Damüls (N.N. 1932).

In the 1930s, the agrarian ecological inheritance of Damüls was superimposed with these facilities, providing services for skiers as well as a labor market for inhabitants. 15 % of the total population were employed in tourism in 1934 (Gross 2012). Samuel Bowles points out that “economic practices […] have long been recognized” by niche construction theorists as impacting biological evolution. “Economic institutions per se constitute a human-constructed environment affecting evolutionary processes […]. Labor markets are persistent and their structures “likely have long-term effects on the process of both cultural and biological inheritance” (Bowles 2000). The development of Damüls shows that the tourism labor market probably had an impact on the village population, because the area would have been entirely depopulated if the tourism labor market had not been growing. The material and institutional construction of a snow-based winter tourism niche in Damüls complemented the existing agro-pastoral niche. This stopped depopulation for the first time since 1880; population grew slightly in the 1930s (Gross 2012).

The ecological inheritance of the agro-pastoral niche in Damüls provided extraordinary sensations when covered at the right time with the right amount of frozen and crystallized water. Sunny, south-facing slopes, cleared by “Walser” settlers allowed skiers to ascend snowy mountain peaks within 2–3 h. The course laid out on the slopes led skiers over grazing areas, both private and common property and used in the summer for dairy farming. In the late 1930s and early 1940s, the agro-pastoral and the tourism niche existed in diachronically, seasonally disaggregated ways (Gross 2012). After farmers had taken cattle from their pasture in autumn and snow covered the Alpine meadow, they shifted from pastoralism to accommodation as well as to instructing and guiding of skiers. In spring, when skiers left Damüls, farmers went back to traditional work. They spread manure, cut grass, produced hay, collected stones from fields, repaired wooden fences and so on (Hessenberger 2013). While novel tourism-related labor practices increased the reproductive success of the inhabitants quickly, inherited property right patterns of land and buildings stabilized wealth over a longer run (Shennan 2011). The inhabitants taught their offspring skills for the tourism and the agricultural labor markets and with it, maximized reproductive success. This can be interpreted as a shift from relocational to counteractive niche construction via social learning, whereby the inhabitants learned to conceptualize the ecological inheritance as a substrate built on frozen water and tourists.

## Stabilizing the tourism niche by ski lifts and a water supply and –purification plant

**Fig. 1:**
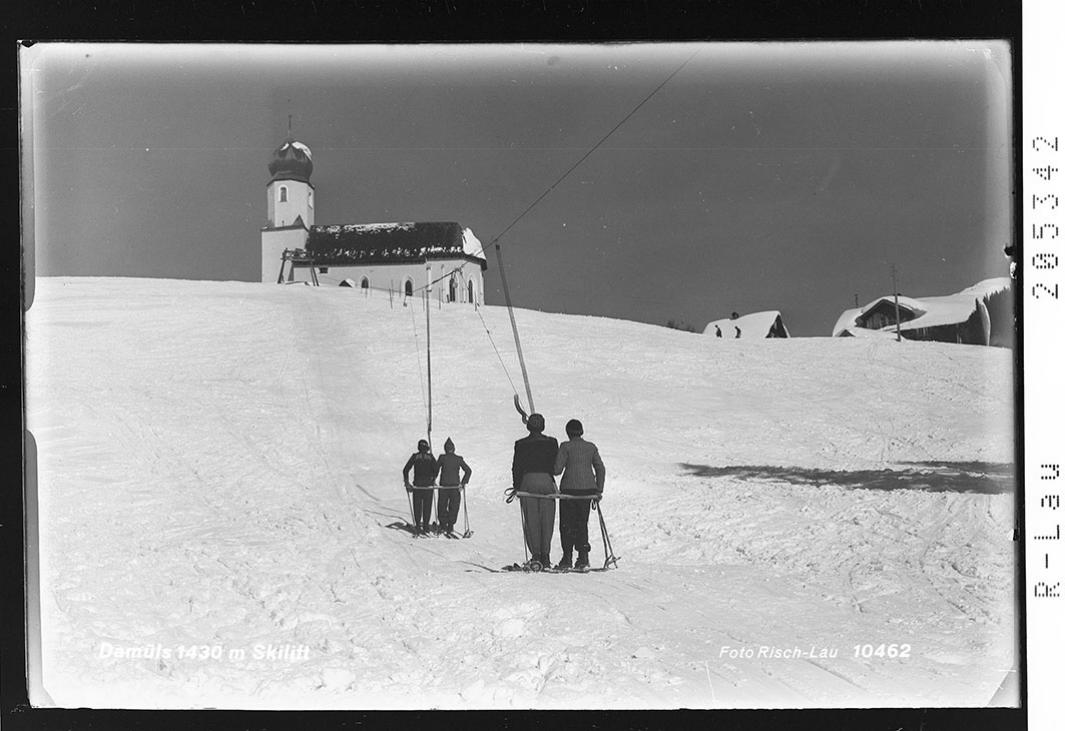
Picture postcard “ski lift in Damüls”; Published by Foto Risch-Lau in 1952. With permission of the Landesbibliothek Vorarlberg

In the early 1940s new plans for winter tourism niches were developed. The ecological inheritance was to be superimposed with ski lifts for a more efficient utilization of the ski slopes. Such technological measures were already in place in stylish winter sport resorts in the Alps (Rigele 2000). Planning authorities, local businessmen and regional politicians assumed that ski lifts would improve attractiveness of the “Wintersportparadies” and thus enhance the reproductive success of the inhabitants (Gross 2012). Ski lifts transported skiers with ease to mountain tops and skiers saved time and physical energy. They accelerated up- and downhill cycles of skiers, allowing them to improve their skiing skills faster than before (Gross et al., forthcoming). Ski lifts could be also very well advertised as a sight, because they worked as popular symbol of modernization in the Alps (Tschofen 1999). It was assumed by tourism entrepreneurs that ski lifts would lead to considerable economic growth in Damüls. The winter sport destination Damüls would catch up with the industrialized zones in the valleys but also with stronger developed resorts within the Alps. Furthermore, they were considered as main agent to fight local unemployment and depopulation. The first T-bar ski lift was built in Damüls in 1947, depicted in Fig. 1

**Fig. 2:**
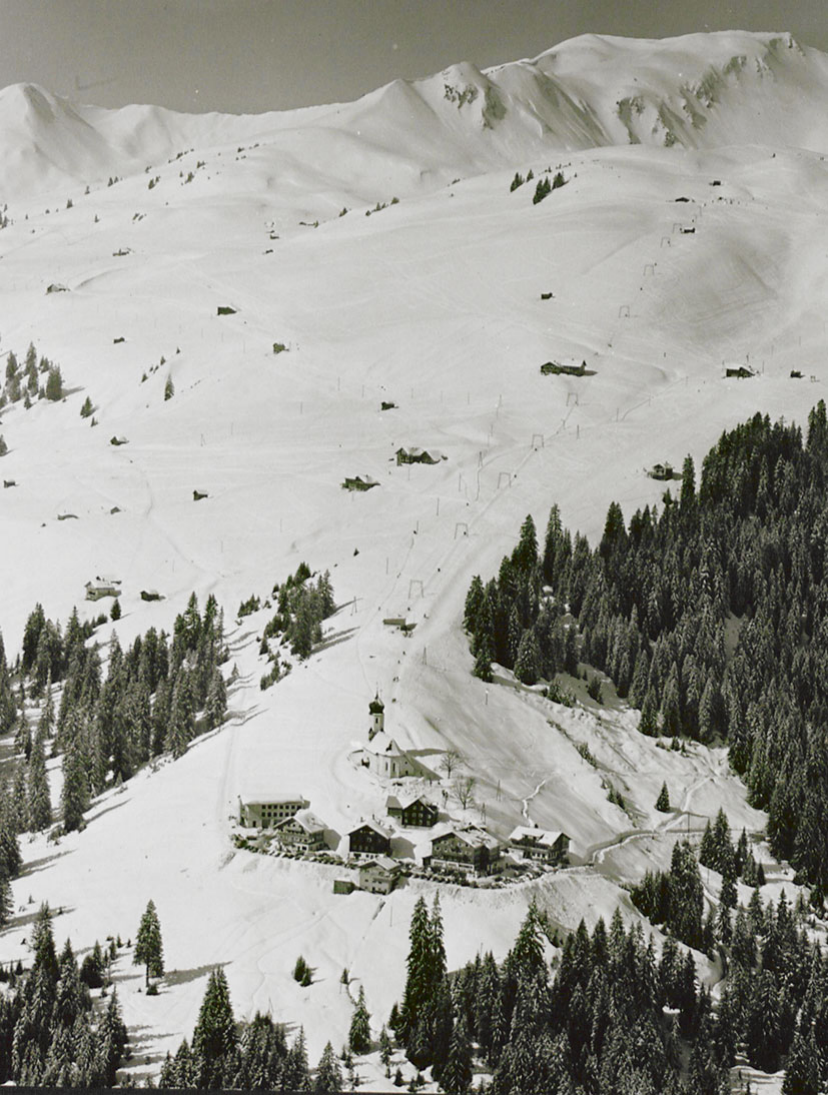
Picture postcard “Schlepplift 1800”; Published by Foto Risch-Lau around 1958. With permission of the Vorarlberger Landesarchiv

The ski lift was situated in the center of the village and managed by tourism entrepreneurs. In the beginning it served primarily as additional offer for the local ski school. The ski lift eased the ascent for greenhorns. It flopped due to its limited length. In 1957, this lift was replaced by another T-bar lift, situated above the church, opening an area up to an altitude of 1,800 m, already known by skiers as perfectly fitting their needs (Gross 2012). The ski lift was named as “Schlepplift 1800” [T-bar lift 1,800 m]. It is depicted in Fig. 2.

In the years after the opening of the ski lift “1800” Damüls underwent a thorough change. Two ski lodges and eight guesthouses were opened by local tourism entrepreneurs shortly after its opening. The accommodation capacity doubled compared to the first tourism boom in the early 1930s. In the early 1960s, 500 beds were available in Damüls (Gross 2012).

The first chair lift was built in 1961, making yet another part of Damüls accessible for skiers. From that point on the ski lift network grew steadily. In only five decades (1961–2010) nine further ski lifts were built (Gross 2012). In the course of the expansion of the ski lift network, many new job possibilities were created. Ski lifts had to be constantly maintained, cashiers—primarily a female job—were needed and a range of unskilled workers were demanded to repair damages on the ski slopes or to help out in hotel kitchens (Hessenberger 2013). The socio-economic censuses reveal a profound shift to a service society. As early as 1970, nearly 70 % of the population worked in the service sector, while 20 % earned their money from pastoralism. In 2001, the share of the service sector increased to more than 80 %. In the same period the number of cattle dropped by about 75 % (Gross 2012). The population of Damüls built up a tourism niche over the second half of the twentieth century, the existence of which depended on the availability of snow for skiing and a growing number of tourists, transported by ski lifts.

The number of winter tourists increased from 3.702 in winter 1948/1949 to 41.542 in 1960/1961 (Vorarlberger Landesregierung Wirtschafts- und Sozialstatistik 1950–1962). One further third of them visited Damüls during the summer. In the early 1960s, drinking and process water supply reached its limits for the first time in the history of the village (VLA Landeswasserbauamt 729/4/27). Until then, households had obtained water from various sources and discharged sewage in individual cesspools. Growth of overnight stays but also day trippers, seasonality of tourism and rising standards outstripped the water supply. At the end of the pipe, the excrement of an increasing number of tourists in the absence of collecting lines and purification plants led to nuisance in Damüls (VLA Landeswasserbauamt 246/3, 5231-2c/48,16). The planning process for a modern water supply and discharge system began in 1961. Water scarcity and sanitary problems were perceived as limiting factors for the tourism niche. The planned water-supply system was fed from the catchment. It featured two connected mains providing drinking water as well as water for the fire brigade (VLA Landeswasserbauamt 729/4/27). Between 1961 and 1970, houses in Damüls were integrated into the water supply system. This was considered as but one measure to sustain tourism growth in the village. More available water soon led to overuse of individual cesspools. Most of them had been designed in the 1940s, when population size, water consumption habits and sewage resulting from tourism had been considerably smaller. In the village center, where hotels and guesthouses agglomerated, the increased amount of sewage flooded cesspools in the high season. Sewage oozed downhill on the surface. During winter, the nuisance was made invisible by the snow cover, but it appeared in spring. In a first step, a drainage system was built in 1961 to channel the sewage. In 1971, a purification plant complemented the drainage (VLA Landeswasserbauamt 246/3, 5231-2c/48,16). An important part of the waters of Damüls had become integrated into a water supply-, drainage- and sewage system that enabled economic reproduction of the inhabitants under circumstances primarily shaped by winter tourism.

## Commodified snow, indebted entrepreneurs and the “ecological inheritance” of agro-pastoralism

Winter season, water, biodiversity and topography became commodities. These habits of commodification were stabilized in economic institutions as well as material objects and enhanced human’s reproductive success in high alpine environments (Bowles 2000). Winter sport destinations were coupled from the very early days to debt-based institutions, incorporating banks, governmental funding organizations and enterprises demanding capital. In 1961, when the ski lift company in Damüls aimed to build a chair lift, they went along the path of debt-based economic growth. From that moment, the ski lift company was obliged to pay back loans and interest regularly (Gross 2013; IOSG 2014). Snow had functioned as selective pressure in the agrarian mode of living but ski lift businesses were literally dependent on snow to pay back loans and interest. This economic constraint, combined with erratic snow fall formed a powerful selective pressure for alpine populations. In reaction to the economic fixation on snow, population in Damüls developed strategies to manipulate snow quality and to handle periods with snow scarcity, thereby introducing a feedback loop reinforcing changes in the ecological inheritance.

**Fig. 3:**
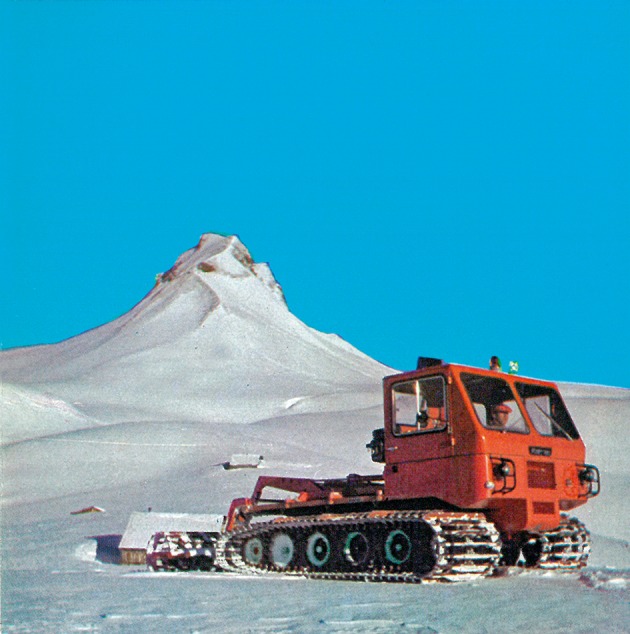
Snow groomer. In: Werbeprospekt “Damüls, die Sonnenterasse in Vorarlberg…”(ca. 1968), pub. by Verkehrsverein Damüls; with permission of Damüls—Faschina Tourismus, Kirchdorf 138, Damüls

The first step into a snow system—a system based on frozen water to mediate selective pressures on the population—was a snow groomer purchased in 1965 (IOSG 2014). Snow grooming leads to snow compaction and slower snow melting. According to a study by the department of conservation New Zealand, snow groomers have several ecological effects: “[S]snow metamorphism is artificially accelerated, and snow density and hardness are increased. […] All this has both thermal and hydrological implications […]. The compaction reduces porosity, permeability and water holding capacity of the mountain slopes, while heat flow rates and length of snow retention are seen to increase” (Fahey and Wardle 1998). Snow groomers create a more stable coverage, less vulnerable to warmer temperatures by impacting snow life-cycle by an artificial aging process. The snow groomer in Damüls was very popular as Fig. 3, published in the tourism advertising brochure of 1968, illustrates.

**Fig. 4:**
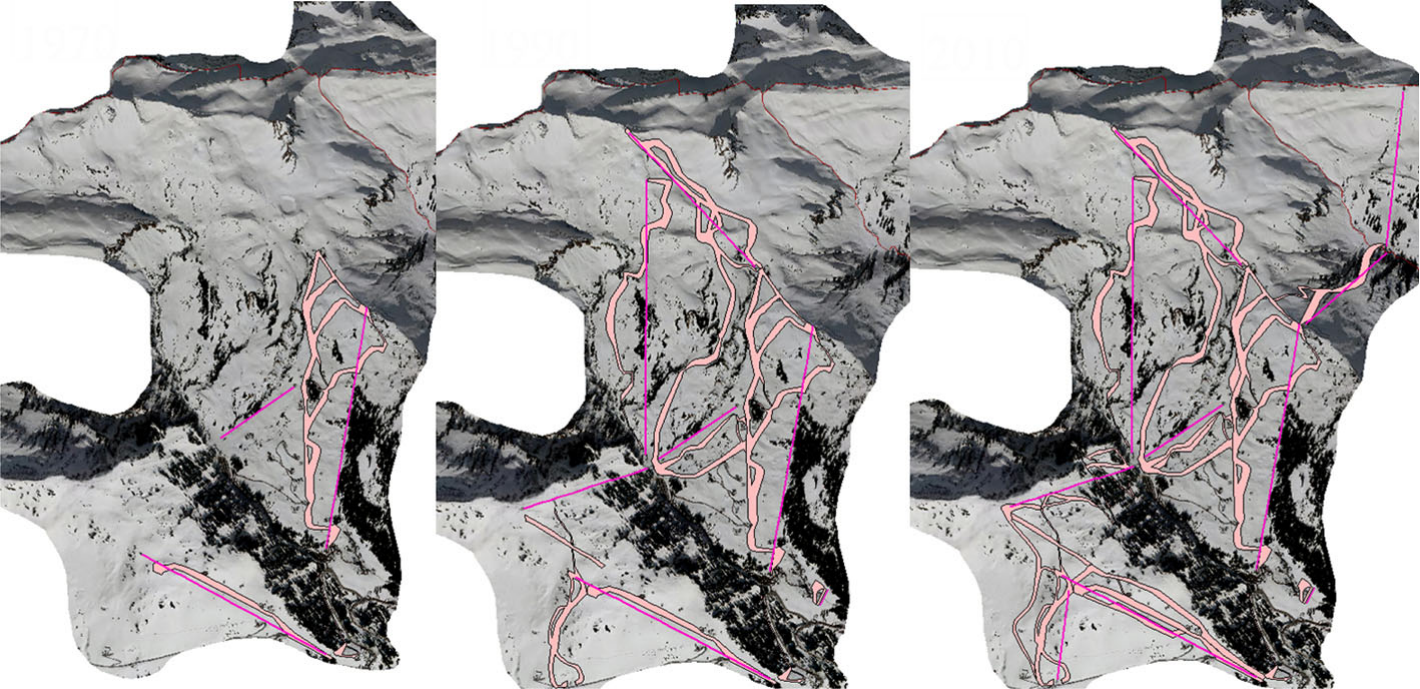
Groomed ski slopes in Damüls in 1970, 1990 and 2010. Map based on aerial photograph from VOGIS and IOSG. Maps by Horst Dolak

The advertorial image of the snow groomer conveys modernity, safety and accessibility. The image signals that ski lift operators mastered to tame the frozen, crystallized water covering the ecological inheritance. Snow groomers and their commercialization as touristic attractions were designed to make the skiing experience predictable and therefore stabilize its profitability. The snow groomer increased the comfort on ski slopes by equalizing different snow qualities. The slopes were easier to handle also by semi-skilled skiers and the ski slopes remained in better condition over the winter season. Both effects together led to a better economic performance of the ski lift company. Driven by a diesel engine, snow groomers can be interpreted as the materialized outcome of a social learning process, which aimed to counteract selective forces by transforming natural snow into a commodity. The maps in Fig. 4 depict the distribution of groomed ski slopes in Damüls:

In 1970 “Lisele” had groomed 19.8 ha of ski slopes. The groomed area has increased to 74.3 ha in 2010. Although this amounted to just 3.5 % of the total area of Damüls in 2010, the groomed ski slopes were a central feature of the tourism niche, stabilizing the tourism labor market. An unwanted side effect of snow grooming is the alteration of species compositions on the grassland. Selective pressures on early flowering plants were increased while the artificially lengthened and densely packed snow coverage provided favorable conditions for plant species adapted to very short vegetation periods (Rixen et al. 2003). Farmers reacted to their enhanced selective pressure, counteracting this technical modification of the agro-pastoral niche when they realized that snow grooming not just altered species composition but also reduced amount and quality of hay. Competing with the tourist niche meant to alter selective pressures for cultivated species in the agricultural niche. To deal with the modification of selective pressures, farmers started to treat slopes with “Thomasmehl”, a substance with a twofold effect. Firstly, it accelerated the melting of snow. Secondly, it compensated the changes in the environment of grassland species. “Thomasmehl” is a budget, black, powdery substance, a byproduct of iron-production, rich in phosphates but also, as should be noticed later, in heavy metals (Bannick et al. 2007). It was used until 1995. Then the use was restricted by law and “Thomasmehl” was replaced by rock meal (IOSG 2014; ICCE 2014).

With a more controlled and predictable skiing experience, ski sports attracted more people, and slopes became more densely packed with skiers. To allow an increasing number of skiers on slopes, slope surfaces and transport capacities had to be extended. Due to strict conservation regulations against slope enlargements from the 1980s onwards, the development of slopes and transport capacities in Damüls followed different trajectories. After 1995, the development of slope area and transport capacity were completely separated. As a result, potential slope area for each skier shrunk drastically between 1970 and 2005, from 86 to 44 m^2^, resulting in an increase of pressure on ski slope routes (Gross 2012). The increased stress on the slopes was particularly acute during poor snow conditions at the end of the season on south-facing slopes (IOSG 2014; ICCE 2014). To deal with this problem and to increase the economic returns of the ski lift company, ski slopes were modified. The slope surfaces were levelled to reduce the dependency of the ski tourism business on high snow covers. Rocks were removed from the slopes and stony, bumpy parts were covered by humus and artificially vegetated with robust plants to stabilize the soil and avoid erosion. Knolls, where snow melted earlier were removed and soil was used to fill dells (Naturschutzanwaltschaft 1976–2012). The aim of these interventions was to create an ideal sloping substrate as carrier of a snow system, which was primarily fed by natural snowfall.

## Water as a limitation for Alpine valley tourism niches

As early as the 1940s, ski resort managers and irrigation equipment suppliers in the United States had begun to experiment with snowmaking. A first patent for such a system was issued in 1954 to Wayne M. Pierce (Mergen 1997). Over the following decades, these systems to lengthen the winter season were rapidly distributed in the United States and subsequently brought to Europe by Fritz Jakob, an employee of the “Linde Group” (Wintersportmuseum 2009). Snow system were engaged to counteract snow absence but also to procure fresh supply when snow had been consumed. When thousands of skiers a day overuse the identical patch of the ski slope, snow disappears, partly by ablation but also by mechanical effects (ICCE 2014). Ski resort managers estimated in the late 1960s “that an average skier displaces 907 kg (1 ton) of snow each day” (Mergen 1997, p. 113). Displaced snow could be partly replaced by snow groomers but some of it was inevitably lost (ICCE 2014).

Negative impacts of snow consumption caused by mass skiing were exacerbated by a change in weather and climate patterns. External pressures by global competition, more and more liberalized markets and high level of debts increased the vulnerability of winter sport operators to the mild winters which had become more common since the 1980s. Each snow-poor winter endangered the economic reproduction of the ski lift and accommodation sector in winter sport destinations. All over the Alps, snow system building boomed in the late 1980s (VLA VIIa-155.06 31.5.1988).

**Fig. 5:**
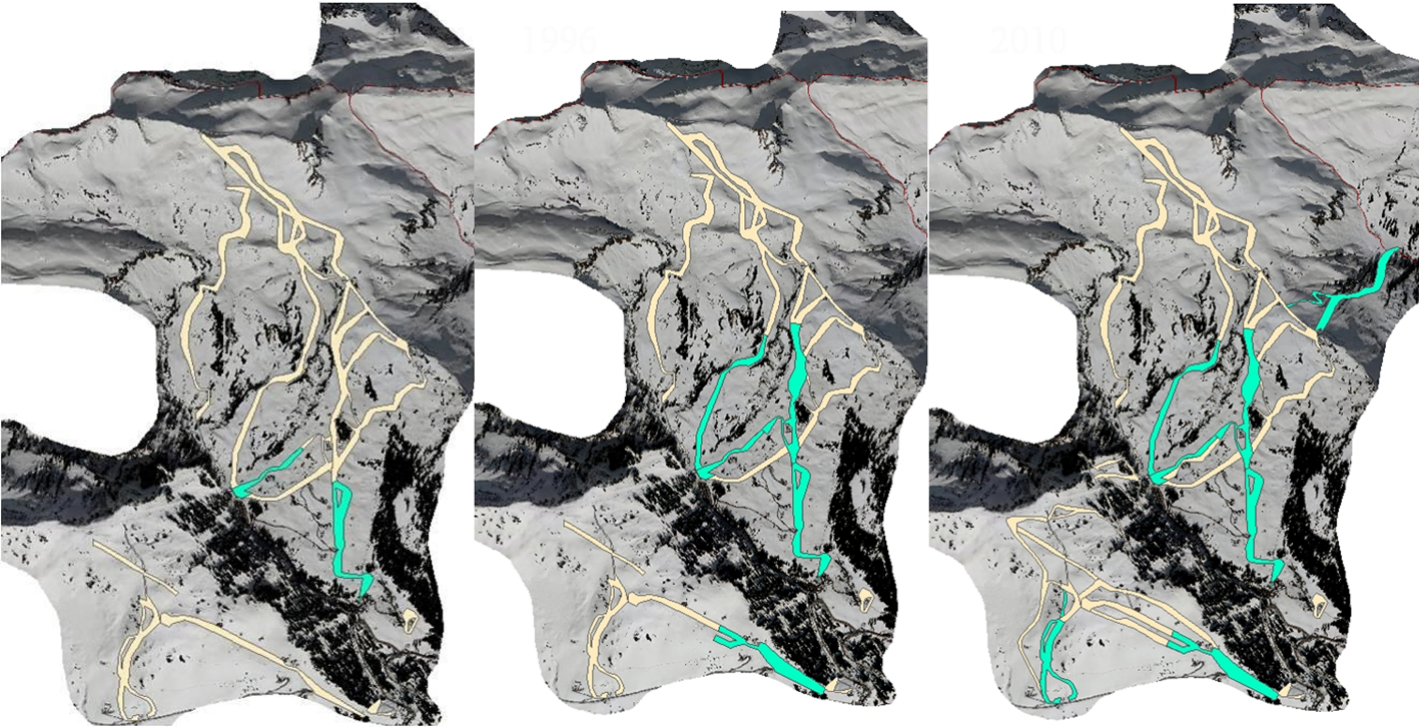
Development of ski slopes covered with artificially produced snow in 1990, 1996 and 2012. Ski slopes covered with artificial snow marked in green, other ski slopes marked in yellow. Map based on aerial photograph from VOGIS and VLA Skipistenbeschneiung Damüls. *Maps* by Horst Dolak

In Damüls, ski tourist numbers in the winter season 1989/1990 declined by about 66 % and the ski lift company suffered an economic loss of about ATS 14 Million (Gross 2012). The ski lift operators had to decide whether to accept income losses and destabilization of the job market or to counteract missing snow by snow making technology. To ensure slope quality throughout the season, the tourism niche was endowed in 1991 with an artificial snow making system, which in that year could deposit 11.920 m^3^ of snow on about 11 % of the slope area (VLA VIIa 422.04). The maps in Fig. 5 depict the share of ski slopes covered with artificial snow (in green) in Damüls in 1990, 1996 and 2012:

Artificial snowing season started in November. When temperature fell below zero over seven nights, the grassland was covered with about 30 cm of snow (ICCE 2014). This artificial snow cover built the foundation on south facing parts of the ski resort, essential for running the ski lifts (VLA VIIa 422.04). In the early years of the snow system, water from the alpine creek Argenbach was sidelined and pumped up several hundred meters to hydrants and snow cannons providing snow for 6.4 ha of ski slopes. 6,7 million liters of water per winter season were dissipated on the ski slopes of Damüls every year between 1991 and 1996 (VLA VIIa 422.04). This equates the daily drinking water amount of Austria’s 8,59 million inhabitants (Austrian Press Agency 2013). In 1996 the area under artificial snow was increased to 9 ha (19 % of the slope area), by 2010, 80 per cent of the slope area was at least temporarily covered by artificial snow (IOSG 2014).

The installation of the snow system in Damüls led to protests of the inhabitants, as they feared noise pollution and further modification of their property. The ski lift company celebrated its 45th anniversary with a small brochure in 1993. It was addressed at the inhabitants of Damüls to promote the benefits of the tourism niche construction activity. The brochure showed a picture of a snow cannon together with a diagram demonstrating the positive effect of snow cannons on tourism in Damüls during periods lacking snow (Damülser Seilbahnen 1993).

**Fig. 6:**
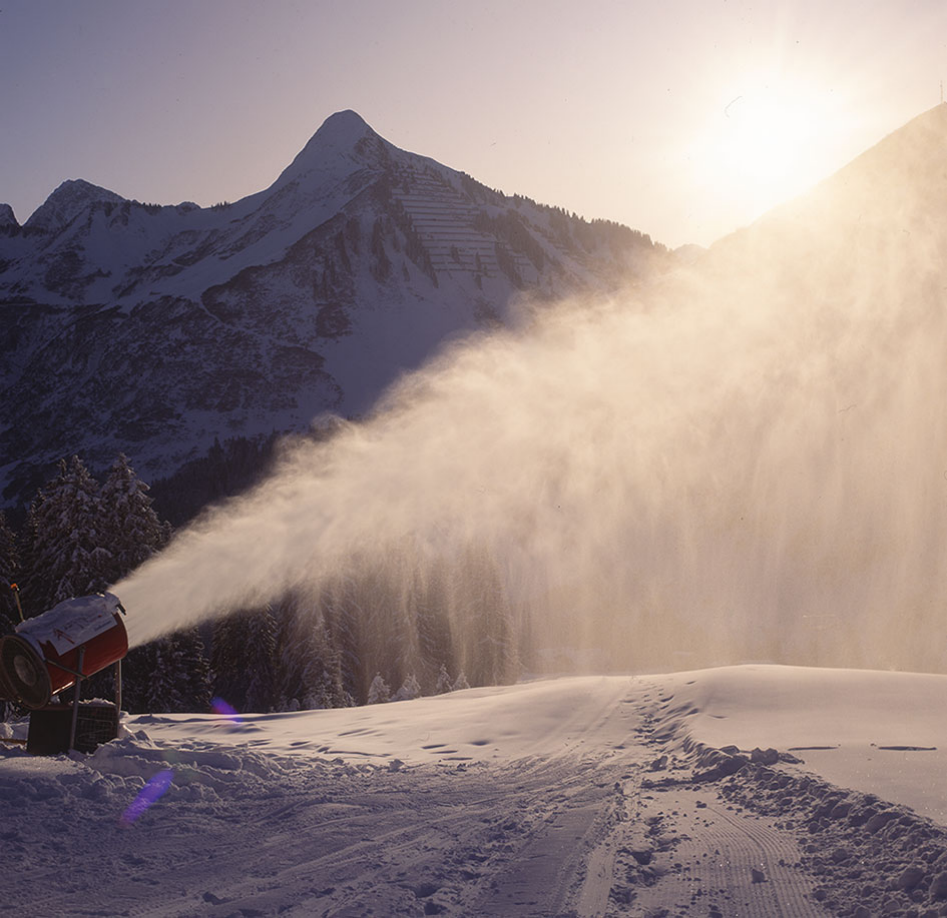
Snow cannon in Damüls. Source: 45 Jahre Damülser Seilbahnen GesmbH and CoKG (1993), with permission of the Seilbahngesellschaft Damüls

Figure 6 shows a snow cannon amidst a high mountain panorama in the middle of a snowy winter landscape. It emanates a brightly lit spray, gently covering the outline of the slope. The background is formed by a beautiful sunrise, the sky bathed in pink, clear and cloudless. All facilities necessary for snowmaking remain invisible, such as the equipment for water disinfection, pipes, cables, pumps, filters, hydrants, as well as the snow groomers. Instead, this picture creates an image of a clean technology in a pristine landscape on an early winter morning, perfectly illustrating the advertising slogan of snow cannon producers “Wasser, Luft und sonst nichts” [nothing else than water and air] (Fachverband für Seilbahnen 1991).

The snow system in Damüls was fed by creek water from the Argenbach. The water was sidelined in low-water periods from a creek that provided habitat to insects, fishes and amphibians. According to an expertise provided by the provincial environmental protection agency, large scale water withdrawal would have been disturbing current, turbulence and characteristics of sediments of the stream. Reproductive cycles of fauna would be influenced. Ecologists foresaw that populations would be diminished and some species would disappear. To minimize selective pressures on aquatic biocoenosis, water removal had to be limited (VLA VIIa 422.04). The water diversion for artificial snowing was also considered as problematic during the low-water periods because the river served as carrier for the outflow from the purification plant in Damüls (VLA VIIa 422.04). Water pollution is a very common legacy of winter tourism development. In the village Lech am Arlberg, where a snow system was installed already in the 1970s, the workers controlling the snow system had to wear protective suits to avoid contact with contaminated river water (ICCE 2014; Mergen 1997). It was assumed that artificial snowing could spread harmful germs, leading to diarrhea and other health problems. The administration was conscious of such issues and enacted a law that water for snow systems had to be controlled regularly and if there was any evidence of contamination, it had to be disinfected with ultraviolet radiation (VLA Beschneiungsrichtlinien). In Damüls, water was clean enough but water withdrawal had to be limited to avoid problematic interactions between snowmaking and sewage disposal but also aquatic biocenosis.

In the late 1990s, the winter sport area was locked onto a path of extension of ski lifts, transport capacity and building of further housing (Gross 2012). Therefore, availability of water for snow systems became relevant as selective force for the winter tourism niche. Hundreds of jobs in Damüls depended on the ability to compensate natural snow scarcity by snow cannons. The capacity of the snow system was sufficient to build a foundation for ski slopes but once a foehn period or phase of warmer weather appeared, the legal water withdrawal limitation increased selective forces on the winter tourism niche by water scarcity. In reaction, the ski lift operators began to sideline water from a range of smaller creeks and also used the drinking water supply. In 1999, after a longer period of intense artificial snowing, inhabitants perceived a decreased hydraulic thrust on the water-tap (IOSG 2014). To avoid a drinking water supply crisis in Damüls, the ski lift operator decided to build a storage pond. The storage pond served as a buffer when much water was needed to produce high quantities of snow in a short period. The storage pond, positioned on the highest point of the ski area, could be filled by smaller creeks over the summer. It was also connected to the Argenbach, so it could be filled by pumping water uphill when enough water was available in the Argenbach. By feeding the snow system with water from several sources, the ski lift operators created a resource-buffer that would create an economic benefit and therefore mediate selective forces in the desired manner (IOSG 2014).

Refinement of river water into artificial snow is an expensive procedure. Purchase of snow cannons, pipelines, hydrants and pumping stations was very costly. Erection and land leases further increased the costs (ICCE 2014; IOSG 2014). Additionally, huge amounts of electricity were needed to pump water upwards. One snow cannon consumes between 15 and 25 kilowatt-hours to convert 1 m^3^ water into 2–2.5 m^3^ of snow (WSL 2013). Working costs of ski lifts increase considerably when snow systems were installed. Therefore, computerized monitoring was integrated into snow systems to make snowing more efficient. Snow groomers in Damüls and elsewhere were equipped with sensing technologies, constantly measuring snow cover and optimizing snowing activities (ICCE 2014; IOSG 2014). Snow conservation practices led to a redesigned ecological inheritance in Damüls. Between the installation of the first snow cannon in 1990 and 2010, ski lift operators considerably re-constructed plains and slopes following a cost- and water saving logic, made necessary by the snow system technology (Naturschutzanwaltschaft Vorarlberg 1976–2012). But soon after ski lift operators installed the snow system they had to realize that the additional output of water due to artificial snowing destabilized slopes and increased erosion. Careful drainage to collect the melting water in spring was but one strategy to reduce harmful side-effects of tourism niche construction.

The second strategy of ski lift operators focused on plant diversity and vegetation of redesigned slope surfaces. As early as 1978, when the first snow systems were installed in the region, ski lift operators began to counteract erosion and slope destabilization by combining scientific, technical and practical knowledge. While they focused on fast re-greening of the slopes in the early years, without taking plant species into account, their efforts shifted more and more to imitate endemic biodiversity (Manhart 1980, 1982, 1984, 1986, 1988; IPAGE 2014). Engineers, landscape planners, gardeners and ecologists worked together in test areas to study how high alpine species, many of them characterized by slow reproductive cycles, could be bred and bedded out (Manhart 1980, 1982, 1984, 1986, 1988). By trial and error, they identified suitable high alpine species. These plant species should revegetate construction sites quickly within 1 or 2 years. Second, plant species should build capacious, widespread roots and a big root mass to stabilize turf and hinder erosion. Third, ideal plant species should reproduce sustainably over time without affording constant fertilizer inputs. Fourth, only plant species adapted to very short vegetation periods were suitable, since snow systems artificially lengthened snow cover (Manhart 1980, 1982, 1984, 1986, 1988). All in all, ski lift operators aimed to create of a plant ecosystem adapted to the selective pressures of high alpine environment, to mass ski tourism as well as to the hydrological and micro-climatic conditions created by snow systems (IPAGE 2014; ICCE 2014).

Snow systems work as temporary storage for water sidelined from high alpine creeks and various other sources. Annual freshwater consumption of snow systems all over the Alps amounted up to 95 million m^3^ in 2004. This equated the annual mean freshwater consumption of a European city with 1.5 million inhabitants (Hahn 2004). Damüls is only a small spot within the Alps, but like many other winter sport resorts it is affected by similar economic and climatic macro-trends. Over the last six decades, winter months became perceivable drier, while more precipitation was recorded during summer months (Laternser and Schneebeli 2003). Considering that ski resort managers all over the Alps plan to quadruple snow making to counteract scarcer snow over the next decades, researchers are worried about long term side effects of tourism niche construction by snow systems (German press agency 2007). Their main characteristic is that they change water cycles. In 2006, Carmen De Jong estimated that up to 30 % of water is lost through evaporation in snowmaking. De Jong concluded that snow making would contribute to a drying of the Alps with appreciable consequences for lowlands (De Jong 2007). The Alps contribute significantly to the water discharge of two of the most important European rivers, the Rhine and the Danube. Thus, mountains are more and more considered as “vulnerable water towers for the twenty first century” (Messerli et al. 2004). According to De Jong, expanding snow systems as counteractive niche construction will impact existing selective pressures, not only on alpine- but also on lowland populations and their water niches, be it drinking-, industrial-, or process water for agriculture. The consequences of local niche construction will be experienced on a continental scale, with an unequal distribution of benefits and burdens.

## Conclusion

High amounts of rainfall, snow covers causing life threatening avalanches, or groundwater increasing erosion impaired reproductive success of the inhabitants in the agro-pastoral niche in Damüls. The inhabitants adapted to the selective environment via a range of niche construction strategies fitting to both the needs of the population and the challenges of water abundance in liquid and frozen aggregate states. In this paper we could show that the inhabitants of Damüls prior to industrialization had learned to cope with the environment primarily by balancing different types of socio-ecological niche construction. Inceptive socio-ecological niche activities accompanied the colonization of the area and transformed high Alpine forests into meadows. An agro-pastoral niche came into existence which was complemented in the nineteenth century with potato cultivation. Poorer inhabitants applied relocational socio-ecological niche constructions activities to balance scarcities by temporary migration and others, rich in muscle power gained income by carrying goods from the valleys. The inhabitants mediated various selective forces by applying different types of socio-ecological niche construction. They reached a steady state, which was accompanied by a positive feedback loop on population dynamics.

While local consequences of socio-ecological niche construction are the focus of the narrative presented, it has to be born in mind that these activities depended on Damüls being integrated into “large-scale, highly cooperative societies, overseen by complex social institutions […]” (Laland et al., S. 79). Human niche construction theory enables to conceptualize complex social networks as a resource to deal with selective forces on a local scale. In this paper this becomes mostly evident when it comes to tourism, which was another form of inceptive socio-ecological niche construction and brought new ways of economic reproduction to Damüls. Tourists were not only seeking beautiful landscapes and body sensations when skiing, as usually argued by tourism historians (Harrison 2001; Urry and Larsen 2011). Skiers in the 1930s were seeking to expose their bodies to extreme selective environments, which were formerly mostly inhospitable, as this was considered an elitist joy (Sellers 1999). The inhabitants learned to conceptualize skiers as a resource and to construct a tourism niche. Functionally, tourism entrepreneurs compensated negative effects of the selective environment on skiers (e.g. frost) by providing heated space and catering and secured access to their ecological inheritance. By doing so, the inhabitants gained a novel relationship to their agro-pastoral inheritance as biophysical substrate for ski slopes. Subsequently, snow turned in their perception from a nuisance into the most valuable local resource. We could show a “lock-in” effect caused by the established local labor market and the import of technologies developed elsewhere, by which the availability of snow became a pivotal selective force. In terms of socio-ecological niche construction theory, the “lock-in” was accompanied by a positive feedback via money, linking the ecological inheritance with labor market and water in a reinforcing way (Puffert 2002; Cowan 1990). Ski lift operators refined artificial snowing techniques but instead of reducing water consumption, they caused a rebound effect (Binswanger 2001).

These locally established feedbacks in villages such as Damüls impacted on upper stretches of European water courses. They bear the potential to exhibit cascade effects on selective forces on lower stretches, if they are further enlarged. Subsequently, control webs of ecosystems on lower stretches, human agriculture, power production and riverine protected areas, serving for leisure needs of urbanized individuals, could be affected. Using the socio-ecological niche construction framework and coupling it to Hutchinsonian niche concepts, incorporating co-existence and co-development of humans and others species’ niches, would offer the potential for interdisciplinary risk assessment across time and scale. A broader understanding of spatio-temporal interconnectivity between multiple elements is possible.

## Footnotes

1 Netting estimated harvests of 150–180 kg potatoes on 0.1 ha.
